# Bioactive Glass Dressings for Infected Wounds: A Systematic Review

**DOI:** 10.1111/iwj.71097

**Published:** 2026-11-10

**Authors:** Nandesh C. Patel, Thomas J. Howe, Henry A. Claireaux, Julian R. Jones, Arul Ramasamy

**Affiliations:** ^1^ Academic Department of Military Trauma & Orthopaedics Birmingham UK; ^2^ Department of Materials Imperial College London London UK; ^3^ Centre for Injury Studies Imperial College London London UK

## Abstract

Wounds pose a clinical challenge due to their susceptibility to infection and complex healing environments. Wound infection with multi‐drug‐resistant bacteria increases the mortality and morbidity, with new strategies needed to treat them. Bioactive glasses are biodegradable glasses that interact with biological systems by releasing ions such as Na^+^, Ca^2+^ and borate. They are emerging as promising adjuncts in managing infected wounds. This review aims to synthesise the current evidence on the role of bioactive glass in infected wounds. This systematic review was reported as per PRISMA 2020 and PRISMA‐S. Medline, EMBASE and CINAHL Complete were searched using a research librarian designed strategy. In vivo studies were screened in duplicate by two blinded authors and quality was assessed with no limitation on study type. Heterogeneity precluded meta‐analysis. Twenty in vivo animal studies were included. Most studies investigated mesoporous or dense nanoparticles (*n* = 17) in murine models (*n* = 20) infected with *S. aureus* . Overall, bioactive glass preparations had faster wound healing compared to comparators by day 14. They consistently reduced bacterial burden in infected animal wound models, with faster and more complete remodelling resulting in better structural and functional skin regeneration. Despite promising results, certainty of evidence was low. This systematic review demonstrates the promise of bioactive glass wound dressings for infected wounds. Findings complement those showing benefit in diabetic chronic wound healing in addition to bone and joint infection. Further research is required to compare bioactive glass dressings to standard‐of‐care dressings to pave the way for first‐in‐human trials to translate these biomaterials for clinical use.

## Key Points

- Wounds pose a clinical challenge due to their susceptibility to infection and complex healing environments.
- Bioactive glass may have an encouraging role to play in infected traumatic wounds, contaminated surgical wounds, and infected ulcers with delayed healing.
- Animal studies shows promising results towards the use of bioactive glass in infected wounds to complement existing human trials in non‐infected wounds.
- Further research is required to evaluate bioactive glass to current care standards in infected wounds to pave the way for human trials.

**Abbreviations**
AMR: antimicrobial resistance
BALB/c: Bugg Albino mouse strain
C57bl/6: C57 Black 6 mouse strain
CFU: colony‐forming units
CI: confidence interval
CINAHL: Cumulative Index to Nursing and Allied Health Literature
EMBASE: Excerpta Medica dataBASE
GelMA: gelatin methacryloyl
GRADE: grading of recommendations, assessments, development and evaluation
HIF‐1: hypoxia inducible factor‐1
ICR: Institute of Cancer Research Mouse Strain
MRSA: methicillin‐resistant *Staphylococcus aureus*

NHS: National Health Service
NIR: near infrared
NPWT: negative pressure wound therapies
PBS: phosphate‐buffered saline
PEGDA: poly(ethylene glycol) diacrylate
PRISMA: Preferred Reporting Items for Systematic Reviews and Meta‐Analyses
PROSPERO: International Prospective Register for Systematic Reviews
SD: standard deviation
SYRCLE: SYstematic Review Centre for Laboratory animal Experimentation
VEGF: vascular endothelial growth factor

## 1 Introduction

The skin is the largest organ in the human body and it has a key role in the defence against infection by forming a barrier [1]. A wound is defined as a disruption in normal anatomical structure and function of skin. Acute wounds normally undergo an ordered restorative process to return to anatomical integrity and function over a period of 4–6 weeks. Chronic wounds fail to undergo this timely restoration or inadequately restore structure and function [2]. Wounds cause significant morbidity and place a burden on healthcare systems and wider society with the estimated cost to the National Health Service being £6 billion a year [3]. Infection within wounds can be considered a failure of immune response, angiogenesis and tissue remodelling which delays wound healing and increases morbidity through risk of recurrence and wound breakdown [4]. This makes preventing and treating infection a key aspect of wound management [5]. Patients with comorbidities, such as diabetes, often have impaired wound healing which increases their risk of chronic wounds that are difficult to treat and can lead to infection, ulceration, amputation and sepsis [6].

Wound dressings are designed to be in direct contact with wounds to promote their healing effects. Traditional dressings are often made from natural fibres like wool and these are often limited to clean and dry wounds, unable to provide optimal conditions for accelerating the wound healing process [7]. Negative pressure wound therapies (NPWTs) can be used for complex wounds, which promote healing through micro‐deformation, fluid removal and optimising the wound environment; however, use in infected wounds risks complications [8]. Subsequently, novel natural and synthetic polymers have been developed to promote a moist wound healing environment and augment this healing process, including hydrogels, hydrocolloids, foams, films and composite dressings [9]. Dressings can be characterised into passive, interactive and bioactive, with traditional dressings being passive in their action [10]. Interactive and bioactive dressings deliver biological compounds which result in a dynamic interaction between the wound and dressing to enhance the wound healing process [11]. Dressings that release metallic ions such as silver, copper, aluminium have demonstrated a role in wound healing because of their antimicrobial properties. Meta‐analyses report that the benefit of silver ions has significantly improved wound healing in specific wound types, for example, diabetic foot ulcers, suggesting context‐dependent efficacy [12, 13]. However, in vitro studies demonstrate that higher levels of silver release can be cytotoxic, supporting cautious use [14]. Despite this, studies have demonstrated that silver incorporated into bioactive glass used on in vitro stem cell and bacterial cultures have enhanced antimicrobial properties without cytotoxic effects, highlighting the potential clinical benefit of bioactive glass [15, 16, 17].

Antimicrobial resistance (AMR) has been highlighted as a Top 10 global public health threat and economic problem by the World Health Organisation [18]. AMR has been linked to an increase in mortality, cost and length of hospital stay. It often contributes to increased adverse outcomes due to ineffective antimicrobial therapy leading to a delay in treatment and increased toxicity of antibiotics [19]. Furthermore, with growing conflict and migration, there has been a rise in multidrug‐resistant organisms contaminating wounds, causing difficult‐to‐treat infections requiring unique management strategies [20].

A bioactive material is one which stimulates a beneficial biological response from the body, particularly, from the host tissue [21]. Bioactive glasses are biodegradable, typically based on silicate, borate or phosphate glass. Their first clinical applications were as bone graft substitutes, as they bond to bone via formation of a bone mineral‐like layer that forms on their surface in vivo. Bioactive glass granules have been used in repairing bone defects after bone‐tumour removing surgery [22]. As they undergo dissolution, they release ions, which can also have bioactive benefits, for example, doses of soluble silica species and calcium ions are known to upregulate osteogenic genes in osteoblasts [23]. Bioglass particles have been incorporated into collagen matrices for wound healing medical devices [24].

Some researchers have linked bioactive glasses to the promotion of angiogenesis viva in vitro studies [25]. Mirragen and MirraSurg (ETS Woundcare, MO, USA) are fibrous wound dressings made with a borate‐based bioactive glass named 1393B3. Amstrong et al. conducted a randomised control trial (RCT) on the use of borate bioactive glass in non‐healing non‐infected diabetic foot ulcers [26, 27]. They demonstrated that their borate‐based bioactive glass fibre matrix led to faster healing of diabetic foot ulcers compared to standard of care with collagen alginate. While they actively excluded infected wounds, this demonstrates the current clinical efficacy of bioactive glass in human wounds. A recent systematic review by Claireaux et al. found a promising role for the use of bioactive glass in the treatment of osteomyelitis and infected non‐union bone fractures, although there remains a lack of RCTs looking at its efficacy in infection in humans [28].

A further benefit of glasses over biodegradable polymers is that these ions can be released at a sustained and predictable rate, which helps reduce the risk of cytotoxicity, a known risk of silver dressings. Researchers have also found benefit in using the glasses for delivering other ions in a range of applications (Figure 1, adapted from Claireaux et al. [28]): for example, scaffolds or nanoparticles can deliver strontium ions that can regulate bone remodelling (osteoclast and osteoblast relative activity). However, most of these are only at the research stage [29, 30]. The glass dissolution is thought to create a hostile environment for bacteria by increasing the pH and osmotic pressure which kills bacteria, furthermore it has been noted that no bacterial resistance to bioactive glass has been observed [31]. This trait can be helpful in wounds infected with drug‐resistant organisms, an increasing occurrence due to AMR. Ions such as cobalt and copper have well‐documented evidence for promoting angiogenesis but other species ions such as silica, calcium and phosphorus are thought to promote endothelial growth factors [25]. This ability to promote angiogenesis along with antimicrobial properties lends potential benefit in soft tissue wound healing especially in the context of chronic arterial disease [32, 33]. This places bioactive glass as a potential dual‐function dressing targeting both infection and host wound healing addressing the known limitations of current single function dressings which focus on wound healing or infection. We hypothesise that bioactive glass that may play a role in acute infected wounds, for example, through trauma or chronic infected wounds, for example, ulcers.

**FIGURE 1:**
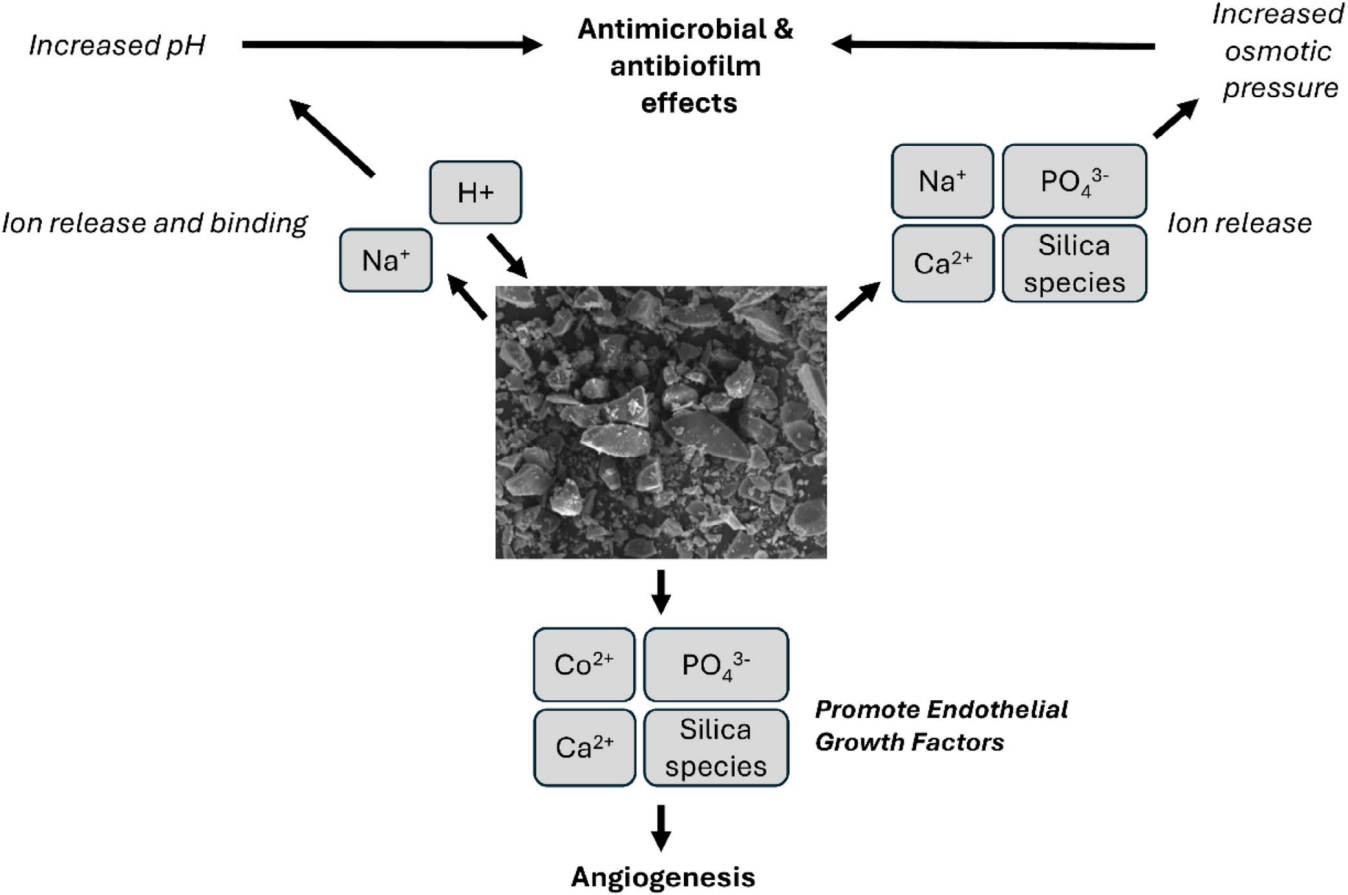
Diagram of mechanistic actions of bioactive glass.

This systematic review appraises the evidence on the use of bioactive glass in the treatment of soft tissue wound infections.

## 2 Methods

This systematic review was reported in accordance with Preferred Reporting Items for Systematic reviews and Meta‐Analyses (PRISMA) 2020 and PRISMA‐S guidelines [34, 35]. The protocol was developed prospectively and registered in the PROSPERO database under protocol CRD420251019674 [36].

### 2.1 Search Strategy

The Ovid interface was used to search EMBASE (1974–27/06/2025) and the EBSCO platform was used to search Medline (1966–27/06/2025) and CINAHL Complete (1937–27/06/2025). The search strategy was produced by a research librarian and was limited to English language with no other limits. Interfaces and exact strategies are provided as Supporting Information. A rapid update search was conducted in February 2026 to ensure inclusion of newly published studies.

### 2.2 Study Selection

All human or animal studies evaluating bioactive glass formulations with/without adjuncts used to treat infected wounds or infected diabetic ulcers, were included with comparison being made to standard wound care. Ex vivo, in vitro studies, conference papers and foreign language studies were excluded. Primary outcome measures were wound closure rates (% at set timepoints) and total bacterial load (reduction in Log10 CFU/g at set timepoints). The secondary outcome measure was wound remodelling (qualitative and quantitative).

Search results were merged and deduplicated using Rayyan (Rayyan, MA, USA) [37]. Abstracts and full texts were screened blinded by two authors (N.C.P. and T.J.H.). Bibliographic screening was performed to identify any articles not captured in our search strategy. Disagreements were resolved by discussion with a third author (H.A.C.).

### 2.3 Data Extraction and Analysis

Two authors (N.C.P., T.J.H.) extracted data independently onto a bespoke spreadsheet. Electronic laboratory notebook was not used. Where numerical data was not available in the manuscripts, this was requested from the corresponding authors via email in October 2025. If there was no response, data was extracted from included graphs using the WebPlotDigitizer tool (v5.2, Rohatgi) as described in the Cochrane Handbook [38, 39]. Other data was analysed descriptively because a lack of comparative or homogenous outcome reporting precluded meta‐analysis.

### 2.4 Quality Assessment

Studies were evaluated against the Animals in Research Reporting In Vivo Experiments (ARRIVE) guidelines 2.0 with a quality coefficient calculated as per the methodology in García‐González et al. [40, 41] The risk of bias was assessed independently by two authors (N.C.P., T.J.H.) using the Systematic Review Centre for Laboratory Animal Experimentation's (SYRCLE) risk of bias tool for animal studies, with figures produced using the Robvis tool [42, 43]. Disagreements were clarified by a third author (H.A.C.). Certainty of outcomes was assessed using the Grading of Recommendations Assessment, Development and Evaluation (GRADE) approach [44].

## 3 Results

Seven hundred and thirteen abstracts and 47 full texts were screened, with 20 studies meeting inclusion criteria (Figure 2).

**FIGURE 2:**
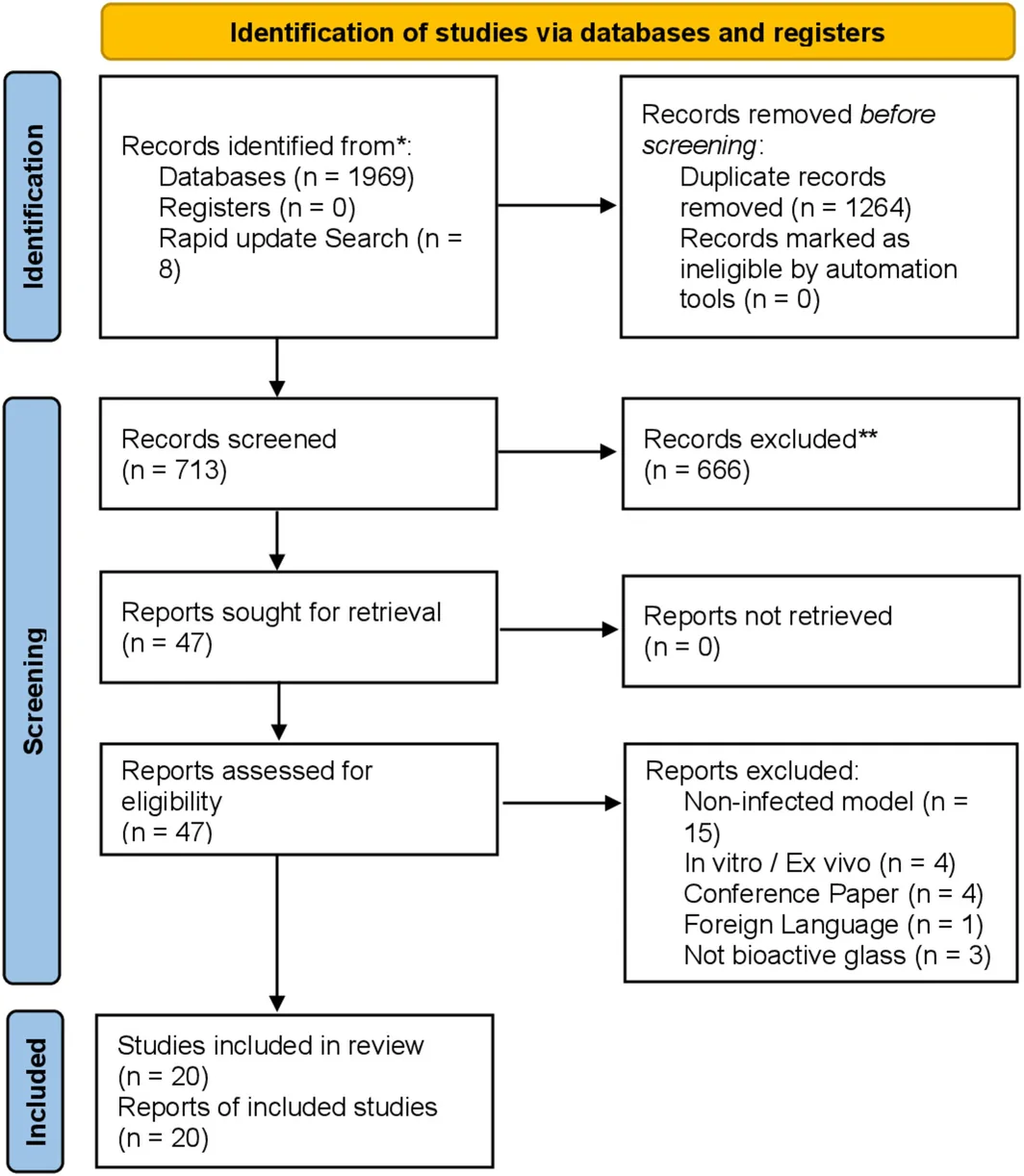
PRISMA flow diagram.

### 3.1 Study Characteristics

Twenty in vivo animal studies were included. Study characteristics and details of animal models are described in Table 1.

**TABLE 1:** Characteristics of included studies.

#	References	Year	Bioactive glass type	Material additive	Carrier	Adjunct	Animal	Species	Sex	Disease model	Concentration of pathogen (CFU/mL)	Comparators
1	Ao et al. [45]	2025	Mesoporous SiO_2_–B_2_O_3_–CaO–P_2_O_5_ particles	Gallium (Ga3^+^)	Bacterial cellulose dressing	—	Rodent	Kunming	Male	ATCC 25923, *Staphylococcus aureus*	1 × 10^6^	Mepilex Ag, bacterial cellulose
2	Fayaz et al. [46]	2024	45S5 dense nanoparticles	Silver (Ag) nanoparticles	Hyaluronic acid/Pluronic hydrogel	—	Rodent	BALB/c	Male	*S. aureus, Pseudomonas aeruginosa*	1 × 10^7^	Hyaluronic acid control, polysporin
3	Guan et al. [47]	2026	Mesoporous SiO_2_–CaO–P_2_O_5_–SrO particles	Tannic acid/Fe	Chitosan hydrogel	Near‐infrared laser (NIR)	Rodent	BALB/c	Male	*S. aureus*	1 × 10^6^	Gauze
4	Hou et al. [48]	2023	Mesoporous SiO_2_–CaO–P_2_O–Fe_2_O_3_ nanoparticles	Iron (Fe)	Hydrogel	NIR	Rodent	Sprague Dawley	Male	*S. aureus*	1 × 10^7^	Tegaderm control, hydrogel
5	Hou et al. [49]	2025	Mesoporous SiO_2_–CuO nanoparticles	Tannic Acid	Phosphorylated GelMA	—	Rodent	C57BL/6	Male	*S. aureus*	1 × 10^7^	Untreated control, GelMA
6	Huang et al. [50]	2022	SiO2–CaO–P_2_O_5_–Fe_3_O_4_ dense Nanoparticles	Ag_2_S	Hydrogel	Near Infrared laser (NIR)	Rodent	ICR	Female	*MRSA*	1 × 10^10^	Untreated control, PEGDA gel
7	Huo et al. [51]	2024	SiO_2_–CuO–ZnO nanoparticles		Porcine gelatin gel	—	Rodent	ICR	Male	*MRSA*	2 × 10^9^	PBS control, Gel, Gel+Zn
8	Jian et al. [52]	2026	Mesoporous SiO_2_–CaO–P_2_O_5_ nanoparticles	Gallium (Ga3^+^), Polyphenol coating	Injectable hydrogel	—	Rodent	Sprague Dawley	Female	*MRSA*	1 × 10^8^	Untreated control, hydrogel
9	Kang et al. [53]	2024	Mesoporous SiO_2_–CaO–CuO–MgO particles		Gelatin‐Tannic acid/PEG gel	—	Rodent	BALB/c	Male	*S. aureus*	2 × 10^8^	PBS control
10	Zhang et al. [54]	2023	SiO_2_–CaO–CuO dense nanoparticles	Alendronate	Pluronic hydrogel	—	Rodent	Kunming	—	*MRSA*	1 × 10^7^	Saline control, Tegaderm, F127@CHO
11	Lin et al. [55]	2024	Mesoporous SiO_2_–CaO–P_2_O_5_ Nanoparticles	Rubidium (Rb) and aggregation‐induced emission luminogens	Composite dressing	—	Rabbits	New Zealand	Female	*S. aureus, Escherichia coli*	1 × 10^8^	Medical gauze control
12	Liu et al. [56]	2023	Mesoporous SiO_2_‐MgO nanofibers	—	—	—	Rodent	C57BL/6	Male	*S. aureus*	2 × 10^9^	Untreated control, 3 M Adhesive Bandages
13	Lyu et al. [57]	2025	SiO_2_—CaO–MgO core with mesoporous SiO_2_–CuO shell Nanoparticles		Carboxymethyl chitosan/poly(ethylene glycol) aldehyde Hydrogel	—	Rodent	ICR	Male	*MRSA*	4 × 10^9^	PBS control, hydrogel
14	Zhang et al. [58]	2023	Mesoporous SiO_2_–CaO–P_2_O_5_ nanoparticles	Polydopamine	Hydrogel	NIR	Rodent	BALB/c	Female	*S. aureus*	1 × 10^2^	PBS Control
15	Miao et al. [59]	2024	Mesoporous SiO_2_‐MgO nanoparticles		—	NIR	Rodent	BALB/c	Male	*MRSA, E. coli*	—	PBS Control
16	Niu et al. [60]	2021	SiO_2_–CaO–P_2_O_5_–MgO dense nanoparticles	Molybdenum (Mo) nanocrystals	Hydrogel	—	Rodent	C57BL/6	Female	*MRSA*	1 × 10^4^	Pluronic F127, Tegaderm
17	Wang et al. [61]	2025	SiO_2_–CaO–P_2_O_5_–MgO Mesoporous nanoparticles		Small extracellular vesicles in hydrogel	—	Rodent	Sprague Dawley	Male	*S. aureus*	7 × 10^8^	Untreated control, Schiff base hydrogel
18	Wei et al. [62]	2022	Mesoporous SiO_2_–CaO–MgO microparticles	Polydopamine	Hyaluronic acid hydrogel	—	Rodent	Sprague Dawley	Male	*S. aureus*	1 × 10^8^	PBS Control, hyaluronic acid gel
19	Zheng et al. [63]	2024	Mesoporous SiO_2_–CaO–P_2_O_5_ nanoparticles	Graphene oxide	Alginate cryogel	—	Rodent	BALB/c	—	*S. aureus*	1 × 10^4^	Untreated control, gel + graphene oxide
20	Zhu et al. [64]	2023	Mesoporous SiO_2_–CaO–P_2_O_5_ nanoparticles	Polydopamine and MnO_2_	Lyophilised GelMA	—	Rodent	Sprague Dawley	Male	*S. aureus*	1 × 10^8^	Untreated control, gel

Across the 20 studies, infected wound models were consistently created using full‐thickness punch biopsies, commonly 7–10 mm in mice, 10–15 mm in rats and 20 mm in a rabbit model. Infective modelling was established by introducing concentrated bacterial suspensions (ranging from 5 to 200 μL) in loads often exceeding 10^7^ colony‐forming units (CFUs) per mL. Infection models all included *Staphylococcus aureus* with 35% (*n* = 7) using resistant strains and 15% adding *Escherichia coli* (*n* = 2) or *Pseudomonas aeruginosa* (*n* = 1). This was done either immediately after wounding or after a short delay (24–48 h) to mimic acute or established infections. A subset of studies aimed to model more chronic wounds by inducing diabetes with streptozotocin, a naturally occurring substance that has specific, rapid and irreversible cytotoxic actions on pancreatic β‐cells [65]. Dressings were applied at the time of infection in acute models, whereas in chronic models these were delayed after confirmation of established infection, with the quantity tailored to fully cover the wound, but the specific quantity of biomaterial applied was often not reported.

Formulations of bioactive glass used usually mesoporous nanoparticles (11/20) or dense nanoparticles (6/20) composed of silica, calcium and phosphate. The glass compositions were often modified with magnesium (6/20), copper (5/20), iron (3/20), gallium (2/20), with others using the additive polydopamine (3/17). The most common form of delivery was a gel (16/20) with other methods including dressings, nanofibers and nanoparticles. Most studies used Phosphate Buffered Saline (PBS) or no treatment as their control or comparator, with others using Mepliex Ag, Tegaderm/3 M dressings, hydrogels, Polysporin or gauze.

### 3.2 Quality of Included Studies

All the included studies are in vivo animal studies and represent Level 5 evidence‐based medicine [66]. The quality coefficients of studies as per ARRIVE 2.0 are summarised in Table 2. Twelve studies were rated as excellent (coefficients between 0.8 and 1) and 8 studies as average (coefficients between 0.5 and 0.8). No studies achieved the maximum coefficient. Using SYRCLE's risk of bias tool for animal studies, all studies were judged to have a high risk of bias (Figures 3 and 4) [42]. Overall the outcomes were judged to have a low level of certainty as per GRADE due to indirectness, inconsistency and risk of bias but showed large, consistent directionality of outcomes to encourage future steps (Table 3).

**TABLE 2:** Quality Coefficients as per ARRIVE 2.0 guidelines.

References	Year	Animal Model	Coefficient	Quality
Ao et al. [45]	2025	Rodent	0.79	Average
Fayaz et al. [46]	2024	Rodent	0.90	Excellent
Guan et al. [47]	2026	Rodent	0.74	Average
Hou et al. [48]	2023	Rodent	0.76	Average
Hou et al. [49]	2025	Rodent	0.69	Average
Huang et al. [50]	2022	Rodent	0.62	Average
Huo et al. [51]	2024	Rodent	0.90	Excellent
Jian et al. [52]	2026	Rodent	0.81	Excellent
Kang et al. [53]	2024	Rodent	0.81	Excellent
Zhang et al. [54]	2023	Rodent	0.86	Excellent
Lin et al. [55]	2024	Rabbit	0.86	Excellent
Liu et al. [56]	2023	Rodent	0.81	Excellent
Lyu et al. [57]	2025	Rodent	0.90	Excellent
Zhang et al. [58]	2023	Rodent	0.71	Average
Miao et al. [59]	2024	Rodent	0.64	Average
Niu et al. [60]	2021	Rodent	0.81	Excellent
Wang et al. [61]	2025	Rodent	0.86	Excellent
Wei et al. [62]	2022	Rodent	0.62	Average
Zheng et al. [63]	2024	Rodent	0.81	Excellent
Zhu et al. [64]	2023	Rodent	0.81	Excellent

**FIGURE 3:**
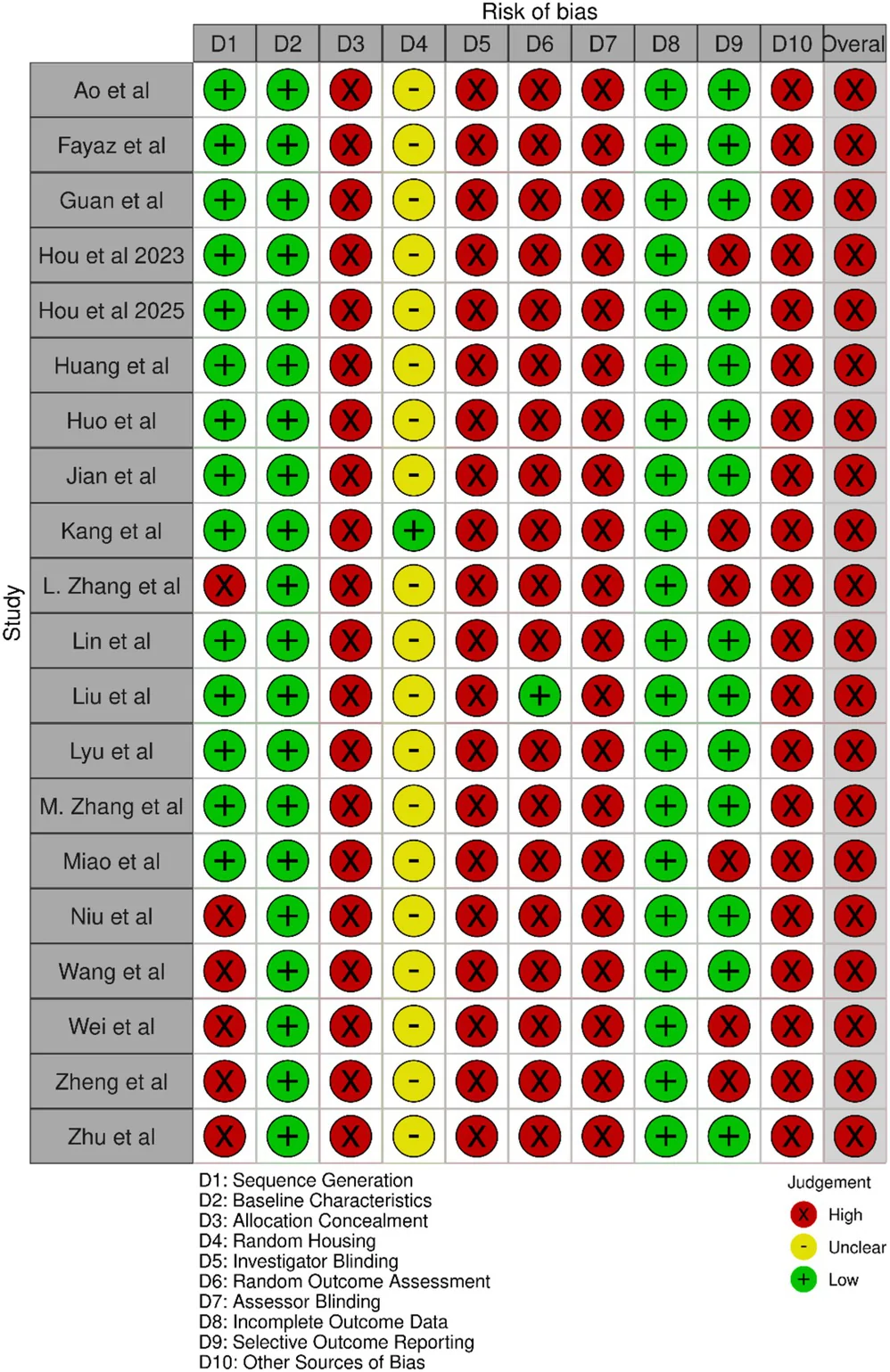
SYRCLE's risk of bias analysis per study.

**FIGURE 4:**
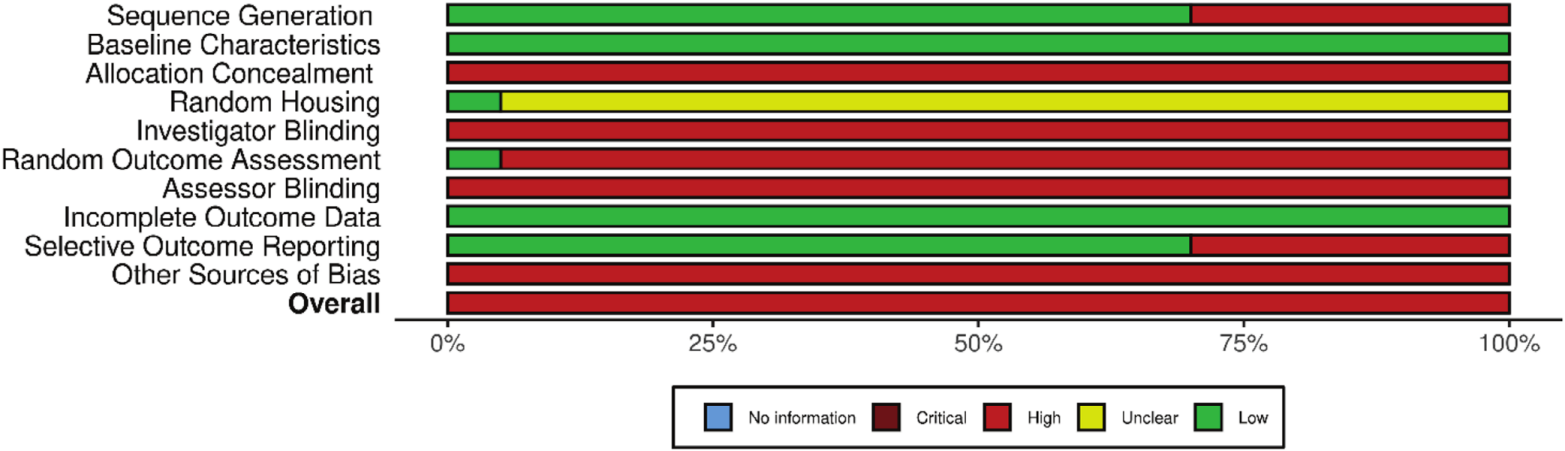
SYRCLE's risk of bias analysis per domain.

**TABLE 3:** Certainty assessment using GRADE approach.

Number of studies	Study design	Risk of bias	Inconsistency	Indirectness	Imprecision	Certainty
*Wound healing*						
20	20 preclinical animal studies	Very serious^a^	Serious^b^	Not serious	Serious^c^	⨁⨁◯◯, low
*Bacterial load*						
9	9 preclinical animal studies	Very serious^a^	Serious^c^	Not serious	Serious^c^	⨁⨁◯◯, low
*Wound remodelling*						
18	18 preclinical animal studies	Very serious^a^	Serious^b^	Not serious	Serious^c^	⨁⨁◯◯, low

*Note:* The symbols are to represent 2/4, and hence a low certainty.

^a^ All studies were assessed to be a high risk of bias as per the SYRCLE RoB tool.

^b^ Although effect sizes were consistent across all three domains, the methods, reporting and groups of preparations assessed were heterogeneous. However, despite this, results remained consistent across all three domains.

^c^ Where reported, CIs are appropriate, however they are seldom reported across many studies. However, all studies do show significance in their data.

### 3.3 Wound Closure

All included studies evaluated the efficacy of bioactive glass treatments in promoting wound healing (Table 4). Across studies, wound closure rates were significantly improved in the bioactive glass‐treated groups. If considering the early healing phase (Days 3–7), Zhu et al. reported an 82.63% closure by day seven in diabetic wounds treated with their cryogels composites of gelatin methacryloyl (GelMA) containing mesoporous SiO_2_–CaO–P_2_O_5_ bioactive glass doped with MnO_2_ nanoparticles and polydopamine (MnO_2_@PDA‐BGs/Gel), compared to 67.55% in the bioactive glass composite cryogel (BGs/Gel) group (no MnO_2_ or polydopamine) and 55.8% in the Gel‐alone group (*p* < 0.001) [64]. Hou et al. found that their phenylboronic acid‐modified gelatin hydrogel containing SiO_2_–CaO–P_2_O–Fe_2_O_3_ nanoparticles applied with near‐infrared radiation (NIR) promoted closure to 62.7% on Day 3 open compared to 38.3% in the Tegaderm control (*p* < 0.001) [48]. Comparable findings were demonstrated by Kang et al. (~85% by Day 7) and Zheng et al. (~60% at Day 4) with bioactive glass dressings consistently outperforming PBS and untreated controls by a minimum of 10% [53, 63]. Guan et al. showed that their CHB10 hydrogel with Mesoporous SiO_2_–CaO–P_2_O_5_–SrO particles had 75.4% and 72.9% healing with and without NIR compared to 44.1% in their gauze control (*p* < 0.001) [47].

**TABLE 4:** Outcome reporting in included studies.

References	Material	Wound closure outcome		Bacterial load outcome		Wound remodelling	
		Day 6	Day 12				
Ao et al. [45]	Ga^3+^@BBG/BC Mesoporous SiO_2_–B_2_O_3_–CaO–P_2_O_5_ particles	58.1% closure^a^	99.8% closure^a^				
	Control	47.5% closure^a^	79.1% closure^a^				
		Day 7	Day 11	Day 3	Day 11		
Fayaz et al. [46]	PL&HA/BG‐AgNPs (45S5 dense nanoparticles)	51.3% closure^a^	100% closure^a^ ^,^ *	5.5 CFU/g^a^ ^,^ *	0.1 CFU/g^a^ ^,^ *		
	Control	37.2% closure^a^	84% closure^a^ ^,^ *	7.1 CFU/g^a^	~1.3 CFU/g^a^		
		Day 7	Day 11			Day 11	
Guan et al. [47]	CHB10 (mesoporous SiO_2_–CaO–P_2_O_5_–SrO particles)	+NIR: 75.4% −NIR: 72.9% Wound closure***	+NIR: 94.3% −NIR: 93.5% Wound closure***			+NIR: 66.0 mm epidermal thickness^a^ −NIR: 45.2 mm epidermal thickness^a^	
	Control	44.1%	78.7%^a^			20.4 mm epidermal thickness^a^	
		Day 7	Day 14			Day 14	
Hou et al. [48]	GTB5 (GelBA + TA + Fe‐BG, mesoporous SiO_2_–CaO–P_2_O–Fe_2_O_3_ nanoparticles)	+NIR: 38.3% −NIR: 46%^a^ Wound area***	± NIR: 0.5%**			+NIR: 65% −NIR: 59% Collagen deposition^a,^ ***	
	Control	62.7% wound area	6.4% wound area			43% collagen deposition^a^	
		Day 3	Day 14			Day 14	
Hou et al. [49]	GP@MBG‐Cu‐TA (mesoporous SiO_2_–CuO nanoparticles)	4% wound area^a^ ^,^ ***	1% wound area^a^ ^,^ ***			Fastest return to normal epidermal structure	
	Control	19% wound area^a^	4% wound area^a^				
		Day 7	Day 14	Day 14		Day 14	
Huang et al. [50]	PBFA (BGN‐Fe‐Ag_2_S + PEGDA, SiO_2_–CaO–P_2_O_5_–Fe_3_O_4_ dense nanoparticles)	+NIR: 34.34% wound area**	+NIR: 3.34% wound area**	+NIR: 0% bacterial viability**		+NIR: Thickest granulation tissue and collagen, highest skin integrity	
	Control	44.49% wound area	16.09% wound area	100% bacterial viability			
		Day 7	Day 14	Day 4	Day 10	Day 4	Day 14
Huo et al. [51]	PADM@CZ (SiO_2_–CuO–ZnO nanoparticles)	51.8% wound area^a^	7.1% ± 7.5% wound area***	2.2 Log_10_CFU/g^a^ ^,^ ***	1.3 Log_10_CFU/g^a^ ***	21% collagen volume^a^ ^,^ ***	70% collagen volume^a^ ^,^ ***
	Control	79.4% wound area^a^	14.1% ± 2.33% wound area	4.7 Log_10_CFU/g^a^	3.2 Log_10_CFU/g^a^	12% collagen volume^a^	37% collagen volume^a^
		Day 7	Day 14			Day 7	Day 14
Jian et al. [52]	PGGBH Mesoporous SiO_2_–CaO–P_2_O_5_ nanoparticles	PGBGH: 88.9% GBGH: 72.1% Wound closure^a^	PGBGH: 97.7% GBGH: 92.0% Wound closure^a^			PGBGH: 1.69 mm GBGH: 1.44 mm Tissue thickness^a^ ^,^ ***	PGBGH: 2.02 mm GBGH: 1.64 mm Tissue thickness^a^ ^,^ ***
	Control	36.3% wound closure^a^	69.2% wound closure^a^			0.89 mm tissue thickness^a^	1.05 mm tissue thickness^a^
		Day 7	Day 15	Day 1	Day 5	Day 15	
Kang et al. [53]	GEL/BG@Cu + Mg (mesoporous SiO_2_–CaO–CuO–MgO particles)	14% wound area^a^ ^,^ **	1% wound area^a^ ^,^ **	18.4% bacterial survival**	6.7% bacterial survival**	81% collagen deposition^a^ ^,^ * Better collagen alignment (0.35)**	
	Control	54% wound area^a^	15% wound area^a^	100% survival^a^	100% survival^a^	44% collagen deposition^a^ Worse collagen alignment (0.87)	
		Day 3	Day 7	Day 3	Day 7	Day 7	
Zhang et al. [54]	FABA (F127‐CHO + BGNCu@AL, SiO_2_–CaO–CuO dense nanoparticles)	71% wound healing^a^ ^,^ *** No pus	94% wound healing^a^ ^,^ ***	19% relative quantity of MRSA^a^ ^,^ ***	0.02% relative quantity of MRSA^a^ ^,^ ***	Better dermal/epidermal restoration	
	Control	19% wound healing^a^	71% wound healing^a^	100% relative quantity of MRSA^a^	100% relative quantity of MRSA^a^		
		Day 10	Day 18	Day 10		Day 18	
Lin et al. [55]	HA‐AMP/SF/Alg/Rb‐BG‐AIEgens (mesoporous SiO_2_–CaO–P_2_O_5_ nanoparticles)	61.5% ± 2.2%	Almost complete closure	Lowest bacterial count		17% collagen deposition^a^ ^,^ **	
	Control	Only reached 61.5% on Day 15		Highest bacterial count		5% collagen deposition^a^	
		Day 7	Day 14			Day 14	
Liu et al. [56]	SiO_2_/MgO‐2% (mesoporous SiO_2_–MgO nanofibers)	39.70% ± 5.57% wound closure*	84.56% ± 9.68% wound closure*			82.69 ± 23.81 μm neo‐epidermis thickness***	
	*S. aureus* + dressing	23.03% ± 6.42% wound closure	66.89% ± 6.99% wound closure			39.08 ± 21.3 μm neo‐epidermis thickness	
		Day 14		Day 4	Day 10	Day 14	
Lyu et al. [57]	CC/AP@CM (SiO_2_–CaO–MgO core with mesoporous SiO_2_–CuO shell nanoparticles)	7.36% ± 0.43% unhealed wound area		2.8 Log_10_CFU/g^a^ ^,^ ***	1.2 Log_10_CFU/g^a^ ^,^ ***	250 μm reepithelialisation +69% collagen volume^a^ ^,^ ***	
	Control	55.30% ± 0.34% unhealed wound area		4 0.3 Log_10_CFU/g^a^	3.1 Log_10_CFU/g^a^	98 μm reepithelialisation + 33% collagen volume^a^	
		Day 6	Day 12	Day 12		Day 12	
Zhang et al. [58]	NO@CD‐PDA/BG NPs (mesoporous SiO_2_–CaO–P_2_O_5_ nanoparticles)	+NIR: 62%*** −NIR: 40% wound closure^a^	+NIR: 91.7*** −NIR: 62%^a^ wound closure	+NIR: reported no viable bacteria		+NIR: More mature epithelium, dense organised collagen and skin appendages	
	Control	38% wound closure^a^	57.7% wound closure	Reported viable bacteria			
		Day 10				Day 10	
Miao et al. [59]	MMPI (ICG + Mg‐MBG + PDA, Mesoporous SiO_2_–MgO nanoparticles)	+NIR: Fastest and infection‐free healing				+NIR: Enhanced collagen, less inflammation	
	Control						
		Day 7		Day 3	Day 7	Day 14	
Niu et al. [60]	BGN‐Mo6 + ‐Mo (B‐15 M = 15MO:BGN, SiO_2_–CaO–P_2_O_5_–MgO dense nanoparticles)	16% wound area**		0.03 × 10^4^ CFU^a^ ^,^ **	0 × 10^4^ CFU^a^ ^,^ **	More hair follicles, microvessels and thicker epidermis	
	Control	48% wound area^a^		4.5 × 10^4^ CFU^a^	3.9 × 10^4^ CFU^a^		
		Day 3	Day 7			Day 14	
Wang et al. [61]	EPPMO (SiO_2_–CaO–P_2_O_5_–MgO mesoporous nanoparticles)	78% wound closure^a^ ^,^ ***	99% wound closure^a^ ^,^ ***			Higher density ECM, most appendages	
	Control	37% wound closure^a^	82% wound closure^a^			Loosely orientated and lacking direction	
		Day 7	Day 14			Day 14	
Wei et al. [62]	HAMA/MBG@PDA (mesoporous SiO_2_–CaO–MgO microparticles)	+MOXI: 94%* −MOXI: 90% wound closure^a^	±MOXI: 99% wound closure^a^ ^,^ *			+MOXI: 85% −MOXI: 79% Collagen deposition^a^ ^,^ *	
	Control	84% wound closure^a^	97% wound closure^a^			70% collagen deposition^a^	
		Day 4	Day 14			Day 14	
Zheng et al. [63]	SA/GO/MBG (mesoporous SiO_2_–CaO–P_2_O_5_ nanoparticles)	No pus 40% wound area^a^ ^,^ ***	4% wound area^a^ ^,^ *			Near complete and faster epithelialisation, larger collagen matrix	
	Control	Wound area increased (107%)^a^	11% wound area^a^				
		Day 7	Day 14			Day 7	Day 14
Zhu et al. [64]	MnO_2_@PDA‐BGs/Gel (mesoporous SiO_2_–CaO–P_2_O_5_ nanoparticles)	82.32% closure***	100% closure***			85% ECM deposition^a^ ^,^ ***	96% ECM + 75% collagen deposition^a^ ^,^ ***
	Control	50% closure	82.63% closure			38% ECM deposition^a^	81% ECM + 46% collagen deposition^a^

^a^ Estimated from graphical data using WebPlotDigitizer [39].

* *p* < 0.05.

** *p* < 0.01.

*** *p* < 0.001.

In mid to late phase healing, Liu et al. showed that their SiO_2_‐MgO glass fibrous membrane promoted 84.56% ± 9.68% (SD) closure by Day 14 compared to 66.89% ± 6.99% (SD) in the 3 M bandage control group [56]. Similarly, Huo et al. observed only 7.1% ± 7.5% (SD) unhealed tissue by Day 14 in mice treated with their porcine dermal matrix derived hydrogel with SiO_2_–CuO–ZnO glass nanoparticles, compared to 14.10% ± 2.33% (SD) in PBS control (*p* < 0.0001) [51]. Guan et al. found almost complete rates of wound closure in their CHB10 hydrogel ± NIR at 94.3% and 93.5%, respectively, compared to their gauze control (*p* < 0.001) [47]. These findings were also supported by Huang et al. (SiO_2_–CaO–P_2_O_5_–Fe_3_O_4_ nanoparticles in polyethylene glycol diacrylate [PEGDA] gel), Lyu et al. (dense SiO_2_–CaO–MgO core with mesoporous SiO_2_–CuO shell nanoparticles) and Wang et al. (dense SiO_2_–CaO–P_2_O_5_–MgO nanoparticles) [50, 57, 61].

### 3.4 Bacterial Load

Ten studies evaluated the bacterial load within wounds using CFU counting, agar plate imaging and semi‐quantitative staining, for example, Giemsa and immunofluorescence.

All studies showed a statistically significant reduction in bacterial load in infected wound models following treatment with bioactive glass materials over set time periods (5–14 days) (Table 4). Fayaz et al. demonstrated that, when compared to Polysporin, a commonly used antibacterial ointment of Bacitracin zinc and polymyxin B sulphate, their hyaluronic acid‐Pluronic hydrogel containing 45S5 Bioglass dense nanoparticles with or without silver nanoparticles significantly reduced bacterial load [46]. The glass‐loaded hydrogels also improved wound closure compared to the gel alone. Kang et al. demonstrated significant bacterial suppression on Day 5 with hydrogel containing mesoporous SiO_2_–CaO–CuO–MgO bioactive glass particles (6.7% bacterial load versus PBS control *p* < 0.01), suggesting rapid antimicrobial action in the early healing phase [53]. Zhang et al. demonstrated that their Pluronic F127 hydrogel containing alendronate decorated SiO_2_–CaO–CuO glass nanoparticle nearly eliminated bacterial growth by day seven in a MRSA‐infected model, despite the presence of MRSA in all groups on day three [54]. This was supported by Niu et al. which demonstrated a 97% reduction on CFU in the 15:1 molybdenum doped bioactive glass‐ceramics (B‐15 M+) hydrogel group and is significantly greater compared to the Tegaderm control (*p* < 0.01) [60].

### 3.5 Wound Remodelling

Nineteen studies evaluated the impact on wound remodelling, a key indicator of wound repair, of bioactive glass therapies (Table 4) through histological staining (H&E or Masson's trichome), with the glasses consistently supporting enhanced wound remodelling.

Zhu et al. demonstrated their MnO_2_@PDA‐BGs/Gel hydrogel significantly increased the density and quantity of extracellular matrix compared to untreated and gel controls at Day 7 with more regular, mature reepithelialisation and evidence of dermal appendages at Day 14 suggesting a perfect repair [64]. Wounds treated with bioactive glass also showed well organised collagen and restoration of epidermal structure as demonstrated by Huo et al., Kang et al., Zhang et al. and Zheng et al. [51, 53, 58, 63]. Liu et al. found their SiO_2_–MgO bioactive glass membrane produced epidermis that was indistinguishable from normal skin, with hair follicles and sebaceous glands [56]. It was also significantly thicker compared to the normal dressing group (82.59 ± 23.81 μm vs. 39.08 ± 21.3 μm SD, *p* < 0.001), which was also demonstrated by Guan and Jian et al. [47, 52].

### 3.6 Adjuncts

Seven studies used adjuncts alongside their bioactive glass dressings. Five studies investigated the use of NIR therapy and one study augmented their bioactive glass with moxifloxacin, a fluoroquinolone antibiotic. All NIR groups used an 808 nm laser at strengths of 0.5–1.5 W/cm^2^ for between 5 and 10 min. Hou et al. used NIR and compared the effects of their hydrogel (with SiO_2_–CaO–P_2_O–Fe_2_O_3_ nanoparticles) with and without their adjuncts. The NIR adjunct enhanced the benefits of the treatments in the early healing period (Days 3–5) but had comparable outcomes by Days 14–21 [48]. Zhang et al. also found that the addition of NIR to SiO_2_–CaO–P_2_O_5_ nanoparticles significantly outperformed the PBS control with a wound closure of 91.7% versus 57.7% by Day 12 [58]. Guan et al. found significantly better rates of healing in their CHB10 hydrogel (with mesoporous SiO_2_–CaO–P_2_O_5_–SrO particles) compared to their gauze control with and without NIR. Whilst their CHB10 + NIR group had slightly better healing rates, there was no significance between groups at Days 3, 7 and 10 reinforcing the results of their bioactive glass hydrogel [47]. Moxifloxacin as an adjunct was explored by Wei et al. who found its addition to a treatment of SiO_2_–CaO–MgO microparticles had significant benefits during early healing, but these were equivocal by Day 14 [62].

## 4 Discussion

This systematic review identified 20 in vivo animal studies demonstrating a possible therapeutic role of bioactive glass in infected wounds. Overall, faster wound healing was seen with bioactive glass preparations compared to controls including PBS and commercial dressings. They consistently reduced the bacterial burden of wounds, supporting their use as antimicrobial wound dressings. Finally, bioactive glass therapies consistently promoted faster and more complete wound remodelling resulting in better structural and functional skin regeneration, including restoration of hair follicles, vascularisation and dermal‐epidermal continuity.

Although there were no human studies on infected wounds in our review, there are trials which support the use of bioactive glass in humans. In their RCT on bioactive glass in chronic diabetic ulcers, Amstrong et al. demonstrated that in their per protocol population, 73% of those treated with borate‐based fibrous glass matrix plus standard of care healed in 12 weeks versus 42% in the standard of care group that used collagen alginate (*p* = 0.007). Although, Amstrong et al. actively excluded infected diabetic ulcers, their study actively demonstrates therapeutic benefits and a good safety profile of bioactive glass in wound healing in humans, which supports the consideration of future human trials using infected models [26]. Claireaux et al. explored the role of bioactive glass in osteomyelitis and infected non‐union [28]. Their data included observational studies which supported high rates of infection resolution and no studies reporting significant differences in adverse events between bioactive glass and comparison treatments. Their evidence reinforces the safety profile of bioactive glass demonstrated by Armstrong et al. and when considered with our results, further supports the use of bioactive glass in infected soft tissue wounds in humans. Vargas Guerrero et al. explored the use of bioactive glass in diabetic wound healing [67]. They found that bioactive glass had improved wound healing properties in diabetic wounds, alongside better remodelling with increased collagen deposition, organisation and density in wounds treated with bioactive glass supporting the findings in this review. Whilst their work was primarily in vitro and explored the mechanistic role of bioactive glass, it demonstrates a role in wider clinical practice for chronic wounds.

The findings of our review suggest that bioactive glass formulations have good efficacy in wound healing in infected environments. In the early inflammatory stage of wound healing, bioactive glass has been found to promote a shift in macrophages from M1 to M2 phenotypes, which promote the wound healing process [67, 68]. Many included studies incorporated metal ions into their bioactive glass formulations to further enhance the antimicrobial and regenerative effects on wounds. The most common metal ions incorporated into the glass particles in the identified studies were copper and magnesium. Copper has antimicrobial properties as it damages bacterial membranes and creates reactive oxygen species, alongside its wound‐healing properties by enhancing angiogenesis through vascular endothelial growth factor (VEGF) and hypoxia‐inducible factor 10alpha (HIF‐1a) stimulation [69]. This further amplifies the existing mechanisms of bioactive glass promoting fibroblast‐secreted VEGF and basic fibroblast growth factor (bFGF) to promote wound healing in the proliferative stage [70]. Magnesium ions have been shown to have enhanced anti‐inflammatory and bactericidal properties alongside enhancing collagen deposition and fibroblast function making it beneficial for antimicrobial management and wound healing in the remodelling stage, which is an important trait in the treatment of chronic ulcers [71, 72]. Synergistic models with Cu and Mg used by Kang et al. outperformed bioactive glass that delivered a single ion. They demonstrated an improved safety profile as there was dose‐sensitive cytotoxicity in Cu‐only systems which was reduced in a dual‐ion (Cu + Mg) system [53].

Several included studies used NIR as an adjunct to enhance the effects of their bioactive glass preparations by elevating wound temperatures to 45°C–50°C. This adjunct led to enhanced bacterial killing, increased blood perfusion and accelerated keratinocyte migration which enhanced wound healing and remodelling in early phase of healing compared to non‐irradiated or control groups, however late‐phase outcomes in groups using bioactive glass were similar. Although Guan et al. found no significance in between their NIR and non‐NIR bioactive glass groups, for other studies it is difficult to differentiate whether the effect is entirely due to the bioactive glass or the adjunct NIR therapy [47, 49, 50, 58, 59].

Furthermore, a variety of infected animal wound models were used by the studies including resistant MRSA models, diabetic wounds and tumour‐excision wounds. Each of these presents a unique, heterogeneous condition which helps micro‐organisms to thrive. The consistent efficacy of bioactive glass dressings in these pathologically complex wound environments highlights the real‐world potential use of bioactive glass in infected wounds in a variety of clinical settings.

### 4.1 Limitations

Our review is the first to look specifically at the effect of bioactive glass on infected wound models. We used a specific and reproducible methodology to evaluate our clinical question. Substantial heterogeneity existed across bioactive glass compositions, ion‐doping strategies, infection induction methods, bacterial strains and outcome assessments across all our studies. Many studies did not publish complete data sets, which introduces publication bias and limited the pooling and reporting of outcome data within our study. To mitigate this, we contacted authors for this data; no replies were received. This methodological variability precluded quantitative synthesis, which limits the strength and quality of our evidence provided.

We excluded organoid models as they do not fully replicate the physiology or disease as accurately as animal models. However, we do acknowledge the limitations of animal models that may not be a direct comparison for human wound healing. Although the authors do recognise the cost benefits and ethical implications of animal testing. Despite this, our review was limited by research published in this area. All included studies are small animal models which looked at short‐term wound healing outcomes and therefore it would be important to understand longer‐term outcomes including sustained bacterial clearance and functional assessment of the skin.

Our studies primarily focussed on acute bacterial infections rather than chronic wounds, which have biofilms that present a significant barrier to healing. This limits the direct extrapolation of our results to chronic infected wounds. Adjunctive photothermal therapy likely amplified early antimicrobial and regenerative effects, which makes it difficult to interpret the independent contribution of bioactive glass therapies.

Whilst some of our included studies did use standard of care controls such as Tegaderm, there is little robust human data available that directly compares the efficacy and health‐economics of bioactive glass to commercial and common antimicrobial dressings for infected soft tissue wounds.

### 4.2 Future Directions

To facilitate meaningful cross study comparison and meta‐analyses, future experimental programmes should prioritise standardised animal models incorporating clinically relevant pathogens, pre‐specified outcome time points and be transparent to allow pooled analysis and the quality of evidence available to materials scientists and clinicians. Pathogen selection should be explicitly aligned to the intended clinical indication and this should be defined a priori rather than introduced heterogeneously across experiments. Whilst *S. aureus* remains highly pertinent to many skin and soft tissue infections, Gram‐negative organisms and resistant phenotypes may be more appropriate. Where adjunctive therapies are employed, for example, concurrent antimicrobial agents, study design and statistical powering should permit isolation of the effect attributable to the bioactive glass.

Translation into early phase clinical trials will face predictable regulatory, methodological and ethical challenges. Mitigating these challenges requires mechanistic clarity at the preclinical stage and predefined concomitant antimicrobial strategies, focusing on progression from mildly infected to resistant wounds. The selection of clinically meaningful endpoints will also be essential: suggestions include time to wound closure, side‐effects and safety and treatment failure. At all stages, comparison to current standard‐of‐care dressings will be important, including silver, iodine and NPWT. Delineation of the principal mechanisms of action will enable rational optimisation of glass composition and formulation, to amplify the dominant therapeutic effects, whilst ensuring preservation of host compatibility and favourable wound healing.

## 5 Conclusion

In conclusion, bioactive glass therapies have been shown to have significant potential in the treatment of infected wounds. Across 20 preclinical studies, bioactive glass preparations consistently improved wound closure, bacterial clearance and wound remodelling. Their effectiveness extended to complex wound models, including diabetic ulcers, MRSA infections and tumour‐resection wounds, demonstrating broad clinical relevance. Despite these promising results, translation is limited by small animal models, adjunct comparators and a lack of homogenous outcome reporting. Future studies must address these gaps to enable safe, scalable clinical adoption. Overall, bioactive glass preparations hold the potential to be used in many diverse clinical situations to treat infected wounds, helping to address the real‐world problem of AMR.

## 6 Take Home Messages

- Bioactive glass dressings show strong antimicrobial activity, reducing bacterial load in infected wounds without evidence of resistance: an important advantage as multidrug‐resistant infections become more common.
- These materials consistently accelerate wound healing in preclinical models, supporting faster closure and better‐quality tissue regeneration compared with standard dressings.
- Ion release from bioactive glass appears central to its benefits, helping modulate inflammation, promote blood vessel formation and support healthy skin repair.
- Although not yet evaluated in infected human wounds, early clinical studies in chronic ulcers show promising safety and healing outcomes, suggesting strong potential for future clinical use.

## Funding

The authors have nothing to report.

## Ethics Statement

The authors have nothing to report.

## Conflicts of Interest

The authors declare no conflicts of interest.

## Supporting information


**Data S1:** Supporting Information.

## Data Availability

The data that support the findings of this study are available from the corresponding author upon reasonable request.
